# Curcumin Pretreatment Induces Nrf2 and an Antioxidant Response and Prevents Hemin-Induced Toxicity in Primary Cultures of Cerebellar Granule Neurons of Rats

**DOI:** 10.1155/2013/801418

**Published:** 2013-12-22

**Authors:** Susana González-Reyes, Silvia Guzmán-Beltrán, Omar Noel Medina-Campos, José Pedraza-Chaverri

**Affiliations:** ^1^Departamento de Biología, Facultad de Química, Edificio F, Segundo Piso, Laboratorio 209, Universidad Nacional Autónoma de México (UNAM), Ciudad Universitaria, 04510 México, DF, Mexico; ^2^Instituto Nacional de Enfermedades Respiratorias “Ismael Cosío Villegas,” 14080 Tlalpan, México, DF, Mexico

## Abstract

Curcumin is a bifunctional antioxidant derived from *Curcuma longa*. This study identifies curcumin as a neuroprotectant against hemin-induced damage in primary cultures of cerebellar granule neurons (CGNs) of rats. Hemin, the oxidized form of heme, is a highly reactive compound that induces cellular injury. Pretreatment of CGNs with 5–30 **μ**M curcumin effectively increased by 2.3–4.9 fold heme oxygenase-1 (HO-1) expression and by 5.6–14.3-fold glutathione (GSH) levels. Moreover, 15 **μ**M curcumin attenuated by 55% the increase in reactive oxygen species (ROS) production, by 94% the reduction of GSH/glutathione disulfide (GSSG) ratio, and by 49% the cell death induced by hemin. The inhibition of heme oxygenase system or GSH synthesis with tin mesoporphyrin and buthionine sulfoximine, respectively, suppressed the protective effect of curcumin against hemin-induced toxicity. These data strongly suggest that HO-1 and GSH play a major role in the protective effect of curcumin. Furthermore, it was found that 24 h of incubation with curcumin increases by 1.4-, 2.3-, and 5.2-fold the activity of glutathione reductase, glutathione S-transferase and superoxide dismutase, respectively. Additionally, it was found that curcumin was capable of inducing nuclear factor (erythroid-derived 2)-like 2 (Nrf2) translocation into the nucleus. These data suggest that the pretreatment with curcumin induces Nrf2 and an antioxidant response that may play an important role in the protective effect of this antioxidant against hemin-induced neuronal death.

## 1. Introduction

The use of natural products has been a general approach for regulating antioxidant homeostasis in cells. Curcumin is a yellow polyphenol compound found in turmeric, derived from *Curcuma longa* Linn. [1]. Curcumin has shown to be an effective anticarcinogenic, antiviral, antioxidant [2–5], and anti-inflammatory substance in human, cell cultures and animal models [6, 7].

Curcumin acts as a direct and an indirect antioxidant since it scavenges reactive oxygen and nitrogen species [8, 9] and induces cytoprotective enzymes such as glutathione-S-transferase (GST), *γ*-glutamyl cysteine ligase (*γ*-GCL), heme oxygenase-1 (HO-1), among others [10, 11]. Curcumin is able to scavenge hydrogen peroxide, peroxyl radicals, superoxide anion, hydroxyl radicals, singlet oxygen, nitric oxide, and peroxynitrite anion [8]. It has been shown that curcumin induces endogenous antioxidant defense mechanisms by modulating transcription factors such as nuclear factor (erythroid-derived 2)-like 2 (Nrf2) [4], activator protein-1 (AP-1), and nuclear factor kappa B (NF*κ*B) [12]. Nrf2 is maintained primarily in the cytoplasm, where it remains bound to the BTB-Kelch-like ECH-associated protein 1 (Keap1 or KLHL19); Keap1 acts as a receptor of electrophilic compounds and promotes Nrf2 ubiquitination for subsequent degradation by 26S proteasome complex [13, 14]. Modification of Keap 1 by oxidation or alkylation (e.g., curcumin interaction) releases Nrf2 and then Nrf2 translocates into the nucleus where it binds as a heterodimer to the antioxidant responsive element in DNA to initiate target gene expression. Nrf2-regulated genes can be classified into phase II xenobiotic-metabolizing antioxidants enzymes, molecular chaperones, DNA repair enzymes, and anti-inflammatory response proteins [15]. These proteins reduce electrophiles and free radicals to less toxic intermediates whilst increasing the ability of the cell to repair any subsequent damage [1, 10, 15, 16]. In this regard, curcumin is able to induce protection and activate Nrf2-dependent protective responses in cell lines or animal models exposed to oxidative conditions [17, 18].

Hemin, a degradation product of hemoglobin, is released by the lysis of red blood cells in hemorrhagic strokes [19, 20]. This molecule is degraded by the isoforms of the heme oxygenase (HO) system: the inducible isoform HO-1 and the constitutive heme oxygenase 2 (HO-2). The HO reaction decreases levels of prooxidant heme, increases the antioxidant biliverdin, and releases antiapoptotic carbon monoxide (CO) [20]. In addition, hemin is a highly reactive compound and a dangerous molecule related to a wide variety of oxidative mechanisms, most of which include enzymatic reactions [21]. Furthermore, it is also known that hemin itself is redox-active and is able to react with peroxides to produce cytotoxic free radicals and oxidative stress. Moreover, hemin is lipophilic and intercalates into the plasma membrane, which may induce lipid peroxidation, as well as interference with membrane fluidity and function [20]. Also, hemin rapidly depletes astrocytic GSH via a peroxynitrite-dependent mechanism before the induction of cell death [22]. It has been demonstrated that hemin is quickly accumulated and slowly degraded by HO, which causes damage primarily, in rat astrocytes and neurons [23, 24]. In addition, hemin iron-dependent injury is not fully established because iron chelators (phenanthroline and deferoxamine) were not able to alleviate the damaging effects of hemin [22, 23]. Moreover, in astrocytes it was found that antioxidants such as trolox or N-acetyl cysteine were not capable of reducing the damage induced by hemin [23]. Therefore, new strategies are essential to counteract the damage induced by hemin. These strategies may involve the improving of the antioxidant potential of brain cells by stimulating HO-1 expression to enhance hemin degradation (to avoid its participation in redox reactions) and the increasing of nonenzymatic antioxidants such as GSH and another cytoprotective enzymes. In this context, curcumin has a plethora of biological effects such as iron chelating, direct, and indirect antioxidant and hormetin (inductor of mild stress) on animal and cell models [25–28].

Taking into account the antioxidant properties of curcumin and the oxidant-mechanisms involved in the toxicity induced by heme groups, the hypothesis was made that curcumin may be able to attenuate the damage induced by hemin in primary cultures of cerebellar granule neurons (CGNs) of rats. It was found that the pretreatment of CGNs neurons effectively prevented hemin-induced oxidative damage. This protective effect was associated with a significant nuclear translocation of Nrf2 and an increase in enzymatic and nonenzymatic antioxidants.

## 2. Experimental Procedures

### 2.1. Reagents

Curcumin (1,7-bis(4-hydroxy-3-methoxyphenyl)-1,6-heptadiene-3,5-dione, high purity ≥98.5%, catalogue no. ALX-350-028-M050, lot no. L12586) was obtained from Enzo Life Sciences, Inc. (Ann Arbor, MI, USA). Basal Medium Eagle (BME), trypsin, deoxyribonuclease type I (DNAse I), cytosine arabinoside, glutamine, glucose, gentamicin, hemin (catalogue no. H5533, lot no. 110K1094), 3-[4,5-dimethylthiazol-*|*2-yl)]-2,5-diphenyl-tetrazolium bromide, L-buthionine sulfoximine (BSO), manganese chloride, bovine serum albumin (BSA), 5,5′-dithio-bis(2-nitrobenzoic acid), 2-vinylpyridine (2-VP), glutathione reduced form, L-glutathione oxidized form, poly-L-lysine, nitroblue tetrazolium (NBT), 1-chloro-2,4-dinitrobenzene (CDNB), ethylenediaminetetraacetic acid (EDTA), xanthine, xanthine oxidase, *β*-NADPH, and anti-*α*-tubulin antibodies were purchased from Sigma-Aldrich (St. Louis, MO, USA). Trypsin inhibitor, penicillin-streptomycin, trypan blue, fetal bovine, and horse serum were purchased from Gibco (Life Technologies, Grand Island, NY, USA). Tin mesoporphyrin (SnMP) was from Frontier Scientific Inc. (Logan, UT, USA). Monochlorobimane and Hoechst 33258 stain were from Fluka (Sigma-Aldrich). Fluorescein isothiocyanate (FITC) conjugated secondary antibodies were purchased from Jackson Immunoresearch Laboratories (West Grove, PA, USA). Anti-HO-1 antibodies were acquired from Enzo Life Sciences, Inc. 5-(and 6-)Carboxy-2′,7′-dichlorodihydrofluorescein diacetate (carboxy-DCFDA) and fluorescein diacetate (FDA) were purchased from Molecular Probes (Life Technologies). Horseradish peroxidase (HRP) conjugated donkey anti-rabbit or goat anti-mouse IgG was from GE Healthcare Biosciences (Pittsburgh, PA, USA) and Invitrogen (Life Technologies), respectively. Anti-Nrf2 and antiproliferating cell nuclear antigen (PCNA) antibodies were from Abcam (Cambridge, MA, USA). The TransAM ELISA kit for Nrf2 (catalogue no. 50296) and Nuclear extract kit (catalogue no. 40010) were purchased from Active Motif Inc. (Carlsbad, CA, USA). Bio-Rad Protein Assay Dye reagent concentrate was purchased from Bio Rad Laboratories (Hercules, CA, USA). All other reagents were of analytical grade and were commercially available.

### 2.2. Primary Cultures of CGNs

Primary cell cultures were obtained from 7-day-old rat cerebellum as previously described [29–31]. Experiments were performed using cells cultured for 9 days in vitro (DIV). The animals were handled and cared with an agreement to the guidelines of the Normal Official Mexicana for the use and care of laboratory animals (NOM-062-ZOO-1999) and for the disposal of biological residues (NOM-087-ECOL-1995). The protocol was approved by the local ethics committee (FQ/CICUAL/059/13). CGNs were cultured in BME supplemented with 50 *μ*g/mL of gentamicin sulfate, 2 mmol/L of L-glutamine, and 10% heat-inactivated fetal bovine serum. Cytosine arabinoside (10 *μ*M) was added 24 h after plating. Glucose (5 mM) was added to the cultures on 4 DIV. CGNs were maintained at 37°C in a 5% CO_2_ atmosphere. Purity of the cultures using this method is around 95% [31].

### 2.3. Culture Treatments

In order to induce oxidative stress, CGNs were incubated with 5–50 *μ*M hemin in Krebs Ringer medium for 1 h, hemin was removed, and CGNs were incubated with culture medium for 24 h. Concentrated hemin solution (8 mM) was dissolved in 40 mM sodium hydroxide and maintained protected from light. This solution was used to prepare the working solution in 10 mM phosphate buffer, pH 7.4. The effect of curcumin on cell viability was established. Concentrated curcumin solution (10 mM) was dissolved in DMSO and maintained protected from light. This solution was used to prepare the working solution in 10 mM phosphate buffer, pH 7.4. This solution was added directly to the culture medium to reach the desired final concentrations. CGNs were incubated with increasing concentrations of curcumin (0–50 *μ*M) for 24 h. At the end of this time, the viability of cells was measured. In further experiments, the potential protective effect of curcumin on CGNs was determined. CGNs were incubated for 24 h with 5, 10, and 15 *μ*M of this antioxidant before the addition of hemin and its viability was evaluated 24 h later.

### 2.4. Cell Viability Measurement

Colorimetric MTT and FDA fluorescent assays were used to measure cell viability. MTT is reduced to formazan by the activity of mitochondrial dehydrogenases, and the absorbance is directly proportional to viable cells [30]. On the other hand, FDA is a cell permeable probe that esterases of living cells convert it to the fluorescent compound fluorescein. Cells were treated with 12 *μ*M FDA for 5 min at 37°C and after washing fluorescence was quantified in a Synergy HT MultiMode Microplate Reader (Biotek, Winooski, VA, USA) using the following wavelengths filters: excitation 485/20 nm and emission 528/20 nm. Cell viability was expressed as a percentage of MTT reduction or fluorescence emission. Viability of control cells (without treatment) was considered as 100%. The value of cells incubated with different treatments was compared with that of control cells. The correlation coefficient between MTT and FDA methods was also calculated.

### 2.5. Western Blot Analysis

At the end of each treatment, cells were harvested in 50 mM phosphate buffer (pH 7.4) with 0.1% triton X-100. The total amount of protein was determined using the Lowry method with BSA as a standard. Western blot analysis was performed as previously described [29]. Protein (30 *μ*g) was separated on SDS-PAGE and transferred to polyvinylidene difluoride membranes (EMD Millipore Corporation, Billerica, MA, USA). After blocking with 5% nonfat milk in blocking TBS-T buffer (Tris, pH 7.4 containing 0.1% Tween-20), membranes were incubated with anti-HO-1 or anti-*α*-tubulin antibodies at 4°C overnight in TBS-T. Afterward, membranes were washed and probed with horseradish peroxidase-conjugated donkey anti-rabbit or goat anti-mouse IgG for 1 h at room temperature. Bands were detected by chemiluminescence using the Millipore ECL detection kit and revealed on autoradiographic films. Densitometry was performed with ImageJ 1.47 (National Institutes of Health, USA).

### 2.6. Determination of Reactive Oxygen Species (ROS)

The measurement of ROS was performed by using the fluorescent probes carboxy-DCFDA and dihydroethidium as previously described [32]. The compound carboxy-DCFDA is deacetylated by esterases, oxidized by ROS and reactive nitrogen species, and converted to the fluorescent compound 5-(and 6-)carboxy-2,7-dichlorofluorescein (carboxy-DCF), staining the cell cytoplasm with bright green fluorescence. Dihydroethidium is oxidized to ethidium in the cytosol mainly by superoxide anion and is then retained within the cell nucleus because of its interaction with DNA and thus staining the nucleus with bright red fluorescence [33].

After cell culture treatments, both fluorescent probes were loaded in Ringer Krebs solution during 20 min. Cells were examined under an epifluorescence microscope using the fluorescent cubes B-2A/C-excitation 450 to 490 nm and G-2A-excitation 510 to 560 nm from Nikon Instruments Inc. (Melville, NY, USA) for the carboxy-DCF and ethidium detection, respectively [31]. The intensity of fluorescence was measured in five random and different fields per well per condition in three independent experiments, using the NIS Elements Imaging software (Nikon Instruments Inc.).

### 2.7. Measurement of Glutathione Content

#### 2.7.1. Total Glutathione (GSH + GSSG) and GSSG Analysis

GSH and GSSG levels were measured in CGNs extracts using the GSH reductase enzyme method [34]. This assay is based on the reaction of GSH and thiol-mediated which produces the 5,5′-dithio-bis (2 nitrobenzoic acid) (DTNB) to 5-thio-2-nitrobenzoic acid (TNB), detectable at *λ* = 412 nm. The test is specific to GSH due to the specificity of the GSH reductase enzyme to GSH: the rate of accumulation of TNB is proportional to the concentration of GSH in the sample. Briefly, cell extract was diluted 1 : 2 with KPE buffer (0.1 M potassium phosphate, 5 mM disodium EDTA, pH 7.5) prior to the addition of freshly prepared DTNB (2.5 mM) and GSH reductase solutions (250 U/mL). Following the addition of *β*-NADPH, the absorbance (*λ* = 412 nm) was measured immediately at 30 s intervals for 2 min. The rate of change in absorbance was compared to that for GSH standards. The measurement of GSSG in each sample was identical to that used for the measurement of GSH, but with a previous treatment of the sample with 2-VP, which reacts out with GSH.

### 2.8. GSH Reduced Form

GSH levels were measured using monochlorobimane as previously described [35]. The fluorescence was measured using excitation and emission wavelengths 385 and 478 nm, respectively, using a Synergy HT multimode microplate reader.

### 2.9. Activity of Antioxidant Enzymes

GR activity was tested using GSSG as substrate and by measuring the disappearance of NADPH at 340 nm each minute for 3 min. One unit of GR was defined as the amount of enzyme that oxidizes 1 *μ*mol of NADPH/min. GST activity was assayed in a mixture containing GSH and CDNB and measuring the increase of optical density at 340 nm each minute for 3 min. One unit of GST was defined as the amount of enzyme that conjugates 1 *μ*mol of CDNB with GSH per minute. Total SOD activity was assayed spectrophotometrically at 560 nm by a method using xanthine and xanthine oxidase for generation of superoxide anion and NBT as the indicator reagent [28]. The amount of protein that inhibited maximum NBT reduction to 50% was defined as 1 U of SOD activity. All the activities were expressed as U/mg protein.

### 2.10. Immunocytochemical Localization of Nrf2

CGNs were seeded on 12-well plates containing glass coverslips treated with 0.025% poly-l-lysine and grown for 9 days. Curcumin was added for 1, 4, 6, 16, and 24 h or 24 h before hemin treatment. Next, cells were washed with phosphate buffer saline (PBS) and fixed with 4% paraformaldehyde for 15 min at room temperature, permeabilized with 0.5% triton X-100 for 20 min, blocked with 3% BSA-0.5% triton X-100-3% horse serum, and incubated with anti-Nrf2 antibody (in 1% BSA-1% triton X-100) for 2.5 h at room temperature. The coverslips were incubated overnight in the dark at 4°C with FITC conjugated secondary antibody and washed with PBS. A nuclear counterstaining was made with a solution of 0.2 *μ*g/mL Hoechst 33258 stain for 1 min and mounting on a slide using Fluoromount Aqueous Mounting Medium [36]. Inverted fluorescence microscope (Nikon Eclipse TS-100F) was used with B-2A/C filter for FITC fluorescence and UV-2A filter for Hoechst signal. The images were acquired with a Nikon Digital Sight DS-Fi 1 camera. Five random images were taken for each well for condition in three independent experiments. NIS Elements Imaging software (Nikon Instruments Inc.) was used for quantification.

### 2.11. Nuclear Extraction and Nrf2 Binding Activity Assay

Nuclear extracts were prepared from CGNs cells using the Nuclear Extract Kit of Active Motif according to the manufacturer's guidelines. Protein concentration in samples was measured using the Bio-Rad Protein Assay Dye reagent. An ELISA-based assay consisting of an immobilized oligonucleotide containing the ARE consensus-binding site (5′-GTCACAGTGACTCAGCAGAATCTG-3′) was used to measure Nrf2 DNA binding activity. Nrf2 from 20 *μ*g of nuclear extract was allowed to bind to the ARE on 96-well plates. A primary antibody against Nrf2 was then used to detect bound Nrf2. A secondary antibody conjugated to HRP provided a colorimetric readout at 450 nm. Nuclear extracts from COS-7 cells transfected with Nrf2 were included as the positive control. The presence of PCNA and absence of protein were used as a measure of the purity of nuclear extracts.

### 2.12. Statistics

Data were expressed as mean ± SEM. They were analyzed with the software Prism 5 (GraphPad, San Diego, CA, USA) by one-way analysis of variance (ANOVA) followed by Bonferroni multiple comparison test or Dunnett test, as appropriate; *P* < 0.05 was considered significant. “*n*” indicates the number of independent experiments.

## 3. Results

### 3.1. Hemin Induces Cytotoxicity and ROS Production in CGNs

**Figure 1:**
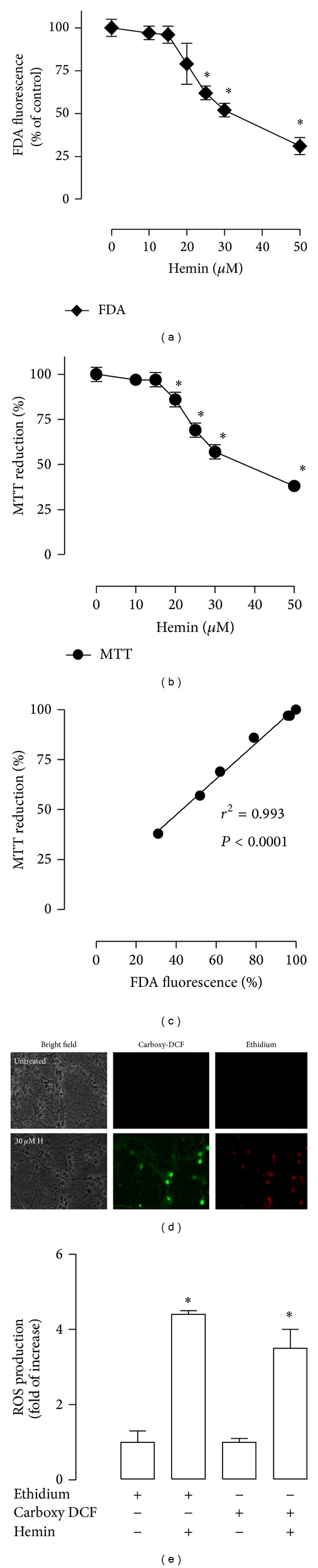
Hemin induced neuronal death and reactive oxygen species (ROS) production in cerebellar granule neurons (CGNs). Cultures were exposed to hemin for 1 h followed by recovery in growth medium for 24 h. The data were obtained after this time. Viability was assessed by (a) fluorescein diacetate (FDA) fluorescence and (b) 3-[4,5-dimethylthiazol-*|*2-yl)]-2,5-diphenyl-tetrazolium bromide (MTT) reduction. (c) Pearson correlation index between FDA and MTT assays. (d) ROS production was evaluated after 1 h of incubation with 30 *μ*M hemin. Bright-field (left panel, H: hemin), 5-(and 6-)carboxy-2′,7′-dichlorofluorescein (carboxy-DCF, middle panel), and ethidium (right panel). The same field is shown in each condition. (e) Intensity of carboxy-DCF or ethidium fluorescence was measured in five different fields per well per condition and was quantified using CGNs with the respective probe as a control. Data are expressed as mean ± SEM, *n* = 3–5. *P < 0.05 versus 0 *μ*M hemin.

It was shown that CGNs exposure to hemin induced a decrease in the viability in a concentration-dependent way from 20 to 50 *μ*M after 1 h of incubation, using two methods: FDA fluorescence and MTT reduction (Figures 1(a) and 1(b)). These methods showed a high correlation (*R* ^2^ = 0.993, *P* < 0.0001) (Figure 1(c)). Incubation with 30 *μ*M hemin for 1 h decreased cell viability by about 50% of control quantified with both assays (*P* < 0.05). Also, cell morphology was verified in bright field micrographs (data not shown). The CGNs treated with the vehicle or 10–20 *μ*M hemin were round and dark with networks of notable processes, but the cells treated with a higher concentration of hemin (30–50 *μ*M) showed morphological alterations, the regular shaped cell bodies seen before were replaced by shrunken, irregular soma and the presence of thin and fragmented neurites. Afterwards, the oxidative effect of hemin was evaluated (Figures 1(d) and 1(e)). Incubation with 30 *μ*M hemin increased fluorescence of carboxy-DCF and ethidium by 3.5- and 4.4-fold (*P* < 0.05), respectively, indicating a marked ROS increase in CGNs (Figure 1(e)).

### 3.2. Curcumin Protects against Hemin-Induced Cytotoxicity and ROS Production

**Figure 2:**
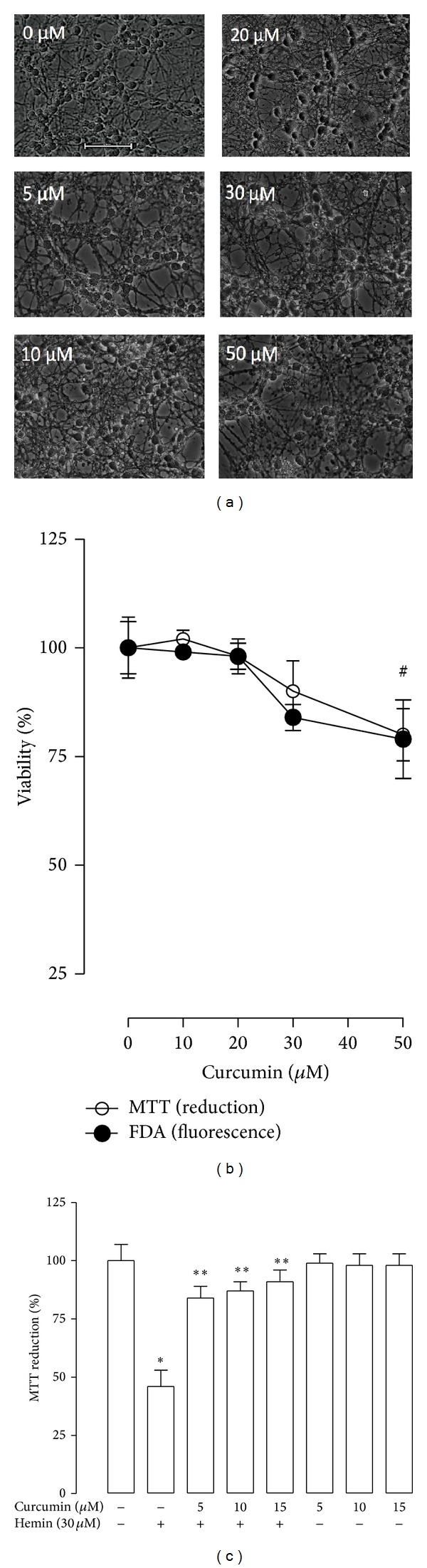
Effect of curcumin on cerebellar granule neurons (CGNs) viability in absence or presence of hemin. (a) Bright field representative images (40x) of CGNs treated with curcumin (0–50 *μ*M for 24 h). Scale bar represents 10 *μ*m and applies to all panels. (b) Viability was quantified by fluorescein diacetate (FDA) fluorescence (∘) and 3-[4,5-dimethylthiazol-*|*2-yl)]-2,5-diphenyl-tetrazolium bromide (MTT) reduction (•). (c) CGNs were incubated with 5, 10, and 15 *μ*M curcumin for 24 h before the addition of 30 *μ*M hemin for 1 h. Subsequently hemin was replaced by fresh medium and the incubation was continued up to 24 h. Finally, cell viability was quantified by MTT reduction and expressed as percentage of control. Data are expressed as mean ± SEM, *n* = 6. ^#^ *P* < 0.05 versus 0 *μ*M, *P < 0.05 versus control (untreated), **P < 0.05 versus hemin.

**Figure 3:**
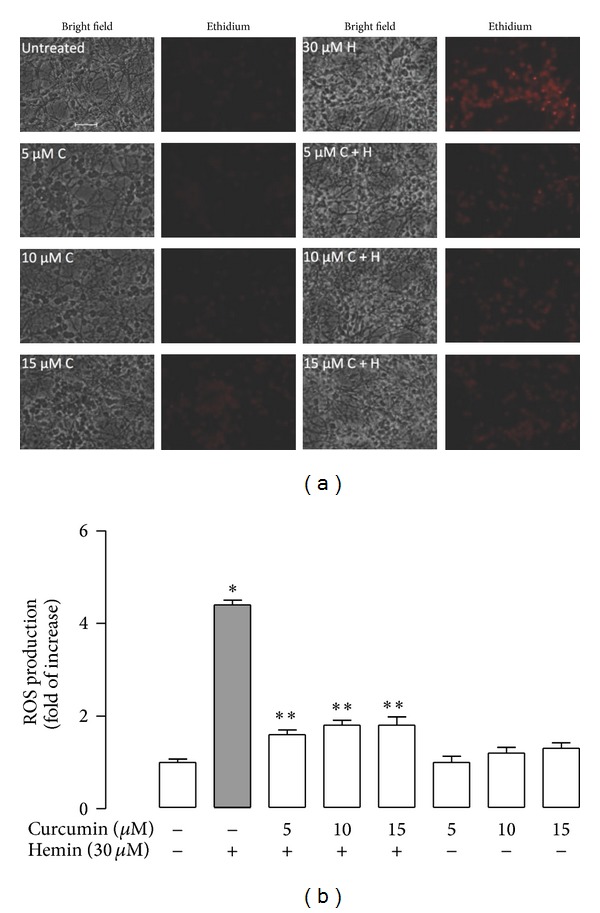
Effect of curcumin (C) pretreatment on hemin (H) induced reactive oxygen species (ROS) production in CGNs. (a) Representative images (40x) after 24 h of treatment with 5, 10, and 15 *μ*M curcumin and exposed to 30 *μ*M hemin for 1 h. Bright-field (left panel) and ethidium fluorescence (right panel). The same field is shown in each condition. Scale bar represents 10 *μ*m and applies to all panels. (b) Intensity of ethidium fluorescence was measured in 5 different fields per well per condition. Data are expressed as mean ± SEM, *n* = 3. *P < 0.05 versus control, **P < 0.05 versus hemin.

Lower concentrations of curcumin (0–40 *μ*M) were unable to induce morphological changes in CGNs (Figure 2(a)). Round and dark cells and a network of processes are prominent throughout the field. Nevertheless, curcumin at higher concentration (50 *μ*M) induced morphological changes such as the presence of thin and fragmented neurites. Cell viability remained unchanged at concentrations ranging from 5 to 30 *μ*M; however, the viability at 50 *μ*M curcumin after 24 h incubation was decreased by 20% and 21% using the MTT and FDA assays, respectively (Figure 2(b), *P* < 0.05). The potential protective effect of curcumin against hemin-induced damage was then assessed. Curcumin significantly decreased hemin-induced cell death in CGNs at all concentrations tested (*P* < 0.05). The percentage of prevention of cell death was 45, 47, and 49 with 5, 10, and 15 *μ*M curcumin, respectively (Figure 2(c)). Moreover, curcumin (5, 10, or 15 *μ*M) was added to the culture 24 h prior to the hemin exposure, and ROS production was measured by fluorometry (Figures 3(a) and 3(b)). It was found that curcumin was able to block the hemin-induced ROS increase (*P* < 0.05) and that curcumin alone slightly increased ROS (Figures 3(a) and 3(b)). Interestingly, the preincubation of curcumin for 1 or 2 h and coincubation of curcumin with 30 *μ*M hemin for 1 h was unable to protect against the hemin-induced toxicity in CGNs on 24 h (data not shown).

### 3.3. Curcumin Increases HO-1 Expression and GSH Levels in CGNs

**Figure 4:**
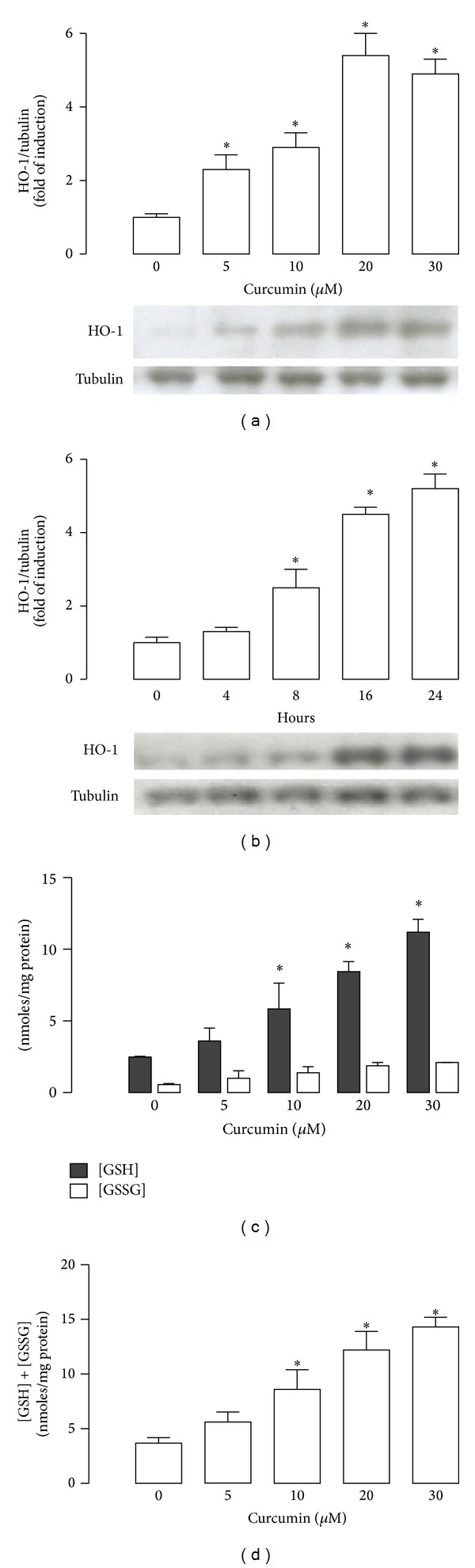
Curcumin increases heme oxygenase-1 (HO-1) protein and glutathione (GSH) and glutathione disulfide (GSSG) levels in CGNs. (a) Curcumin induced HO-1 expression in a concentration (after 24 h incubation) and (b) time-dependent (with 15 *μ*M curcumin) manner. Upper panels show graphs of densitometric analysis (HO-1/tubulin) from each band; lower panels show immunoblot of HO-1 and loading control with tubulin. (c) GSH and GSSG levels were determined 24 h after treatment with 5–30 *μ*M curcumin. (d) [GSH] + [GSSG] content was evaluated 24 h after incubation with curcumin. Data are expressed as mean ± SEM, *n* = 4-5. *P < 0.05 versus 0 *μ*M curcumin or 0 hours.

**Figure 5:**
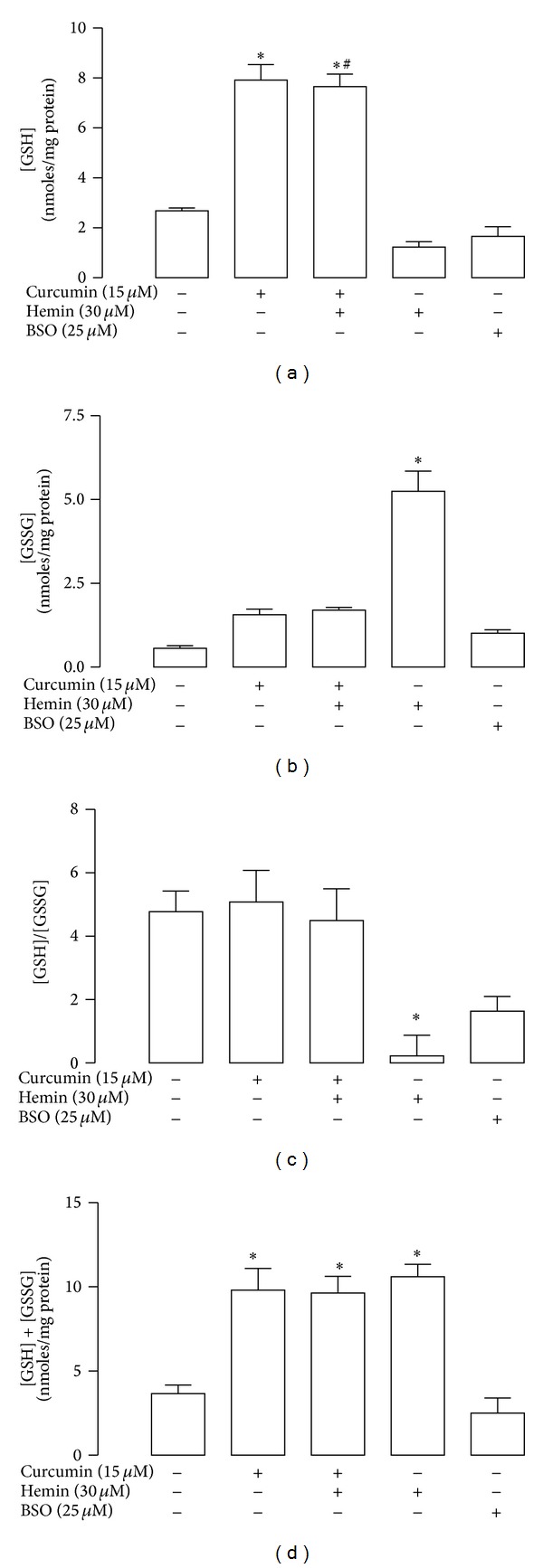
Curcumin prevents hemin-induced changes in glutathione (GSH) and glutathione disulfide (GSSG) levels in CGNs. Cells were incubated with 15 *μ*M curcumin for 24 h. The culture medium was removed and 30 *μ*M hemin was added for 1 h. Hemin was replaced by fresh medium and incubation was continued up to 24 h. (a) GSH levels were assayed with monochlorobimane. (b) GSSG levels were quantified by using 2-vinylpriridine (2-VP). (c) [GSH]/[GSSG] ratio was calculated. (d) [GSH] + [GSSG] content was quantified by 5,5′-dithio-bis(2-nitrobenzoic acid) (DTNB). Buthionine sulfoximine (BSO), an inhibitor of GSH synthesis, was used as a control. Data are expressed as mean ± SEM, *n* = 5. *P < 0.05 versus control (untreated), ^#^ *P* < 0.05 versus hemin.

Curcumin induced HO-1 protein levels in a concentration-dependent manner (Figure 4(a)). Exposure of CGNs to 5 *μ*M curcumin by 24 h increased HO-1 levels by threefold compared to control (*P* < 0.05). The maximum level of expression of 5.4- and 4.9-fold was reached at 20 and 30 *μ*M, respectively (Figure 4(a)). Furthermore, 15 *μ*M curcumin induced a time-dependent increase of HO-1 protein levels starting at 4 h; the increase was significant at 8, 16, and 24 h (Figure 4(b), *P* < 0.05). Moreover, curcumin induced a significant increase of GSH and [GSH] + [GSSG] levels after 24 h of incubation at all tested curcumin concentrations (5 to 30 *μ*M) in a concentration-dependent way (Figures 4(c) and 4(d), *P* < 0.05). GSH levels were also evaluated in CGNs cultures incubated with curcumin for 24 h before hemin treatment. First, curcumin and curcumin plus hemin significantly increased GSH levels (Figure 5(a), *P* < 0.05). Hemin significantly increased GSSG levels (ninefold) (Figure 5(b), *P* < 0.05) and decreased [GSH]/[GSSG] ratio (Figure 5(c), *P* < 0.05). Moreover [GSH] + [GSSG] levels were increased with curcumin alone, curcumin plus hemin, and hemin alone (Figure 5(d), *P* < 0.05).

### 3.4. The Inhibitors of the HO System and GSH Synthesis Abolish the Protection Induced by Curcumin in Hemin-Treated CGNs

**Figure 6:**
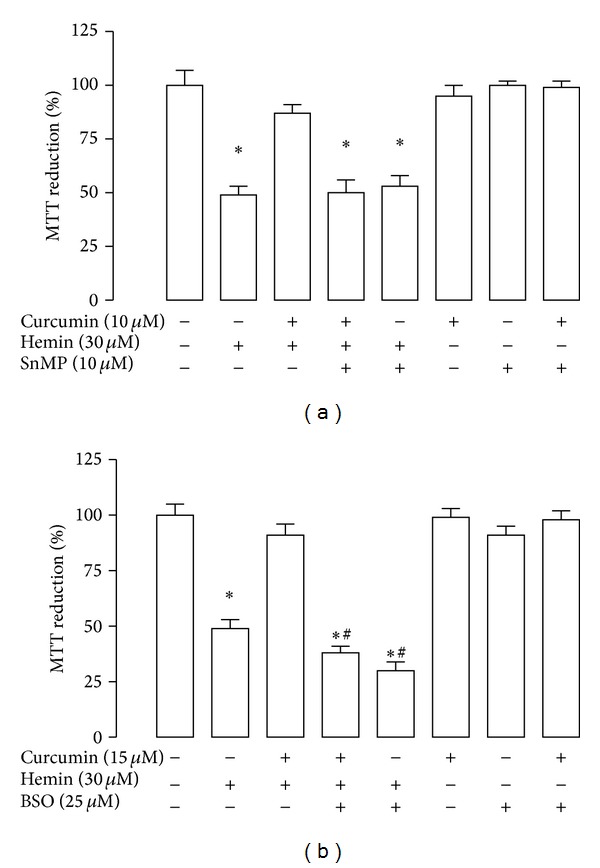
The protective effect of curcumin on hemin-induced cell death was prevented by the enzyme inhibitors tin mesoporphyrin (SnMP) or buthionine sulfoximine (BSO). CGNs were incubated with 10 and 15 *μ*M curcumin for 24 h. The culture medium was removed and the inhibitors (a) SnMP or (b) BSO were added (15 min and 1 h, resp.), before the addition of 30 *μ*M hemin. Subsequently these compounds were replaced by fresh medium and the incubation was continued in presence of inhibitors for 24 h. Finally, cell viability was quantified by MTT reduction. Data are expressed as mean ± SEM, *n* = 6. *P < 0.05 versus control (untreated), ^#^ *P* < 0.05 versus hemin.

For the assessment of the mechanisms by which curcumin-induced protection, the following inhibitors were used: SnMP, an inhibitor of the HO system and BSO, an inhibitor of *γ*-GCL, the rate-limiting enzyme of GSH synthesis. Cells were incubated 15 min with 10 *μ*M SnMP and then exposed to 30 *μ*M hemin. The incubation with SnMP or curcumin alone or SnMP/curcumin had no effect on cell viability. In contrast, it is evident that coincubation with SnMP/curcumin with or without hemin significantly affected the viability of CGNs and blocked cell protection (Figure 6(a), *P* < 0.05). On the other hand, the CGNs pretreated for 24 h with 15 *μ*M curcumin were first incubated with 25 *μ*M BSO for 1 h and then exposed to 30 *μ*M hemin. In this condition, cell death induced by hemin was evidently exacerbated during GSH depletion induced by BSO (*P* < 0.05). Cell viability was unaffected by incubation with BSO or curcumin alone or BSO/curcumin (Figure 6(b)).

### 3.5. Nrf2 Was Activated by Curcumin and Localized in Nucleus in Neuronal Cultures

**Figure 7:**
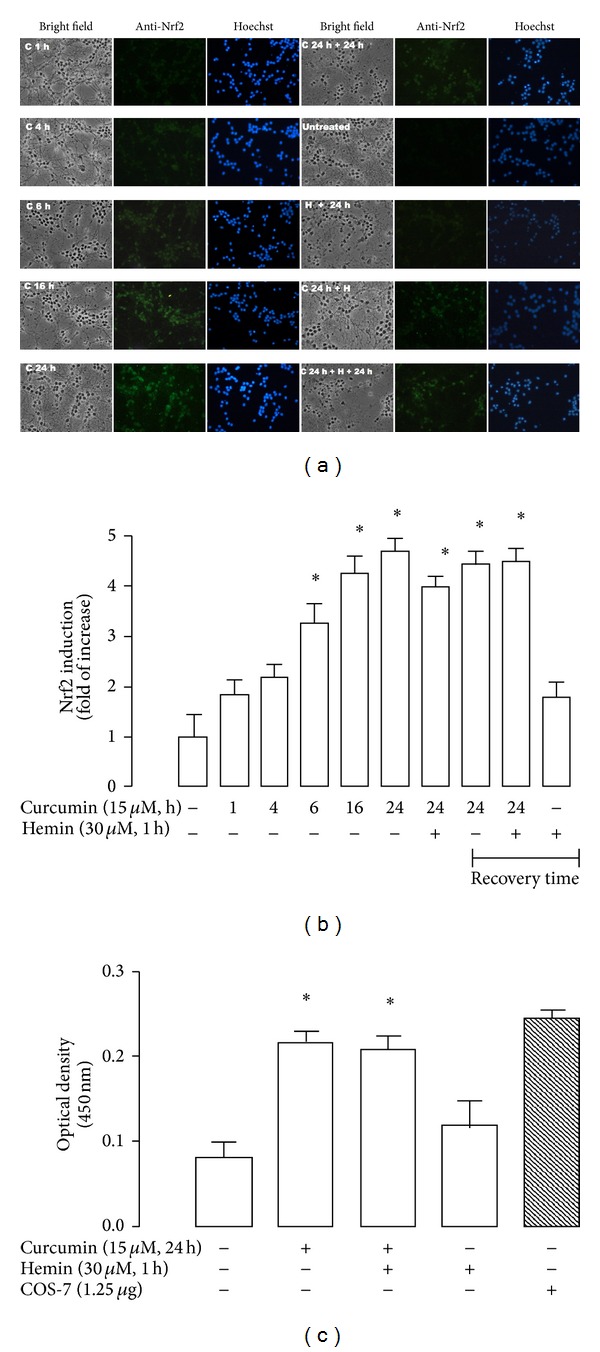
Immunocytochemistry localization and functional assay (antioxidant response element (ARE) binding) of Nrf2 in CGNs exposed to curcumin, curcumin/hemin, and hemin. Curcumin (C) was capable of inducing nuclear translocation and binding of nuclear Nrf2 to ARE before and after hemin (H)-exposure. (a) Representative images with bright-field (40x, left panel), anti-Nrf2 signal (middle panel) and Hoechst stain (right panel). The same field is shown in each condition. CGNs were incubated (1–24 h) with 15 *μ*M curcumin (C 1 h, C 4 h, C 6 h, C 16 h, C 24 h). In addition, cells were pretreated for 24 h with curcumin and then exposed to 30 *μ*M hemin for 1 h (C 24 h + H). Additional conditions were the following: CGNs were incubated by 24 h with 15 *μ*M curcumin and 24 h of recovery was allowed (C 24 h + 24 h), CGNs were incubated by 24 h with 15 *μ*M curcumin, then curcumin was removed, and 30 *μ*M hemin was added by 1 h and removed and 24 h of recovery was allowed (C 24 h + H + 24 h) and CGNs were incubated by 30 *μ*M hemin by 1 h and then removed and 24 h of recovery was allowed (H + 24 h). (b) Intensity of fluorescence was measured in five different fields per well per condition. (c) Curcumin was incubated for 24 h or hemin for 1 h and the transcriptional activity of Nrf2 was measured at 450 nm using immobilized oligonucleotides containing the ARE consensus binding site. COS-7 nuclear extract was used as a positive control of this assay. Data are expressed as mean ± SEM, *n* = 3. *P < 0.05 versus control (untreated) and versus hemin.

Curcumin was able to bring about nuclear translocation of Nrf2 in a time-dependent way. Nrf2 was localized in nucleus after incubation with 15 *μ*M curcumin by 4, 6, 16, and 24 h (C 4 h, 6 h, 16 h, and 24 h, Figure 7(a)). Furthermore, signal of Nrf2 was seen 24 h after 24 h of incubation with curcumin (recovery time) (C 24 h + 24 h, Figure 7(a)).

It is interesting to mention that fluorescent signal of Nrf2 significantly colocalizes with nuclear signal at 16 and 24 h of incubation (Figure 7(a)). Also, the signal of Nrf2 was shown in cells treated with curcumin (24 h of incubation) and hemin (C 24 h + H, Figure 7(a)) and 24 h of recovery time (C 24 h + H + 24 h, Figure 7(a)). Hemin with or without 24 h of recovery time (H + 24 h) induced a nonsignificant accumulation of Nrf2 in the nucleus, but when curcumin was preincubated, strong levels of induction were observed (Figure 7(b), *P* < 0.05). Additionally, using the TransAM ELISA kit, the nuclear Nrf2 binding activity was assessed in CGNs from control, and 15 *μ*M curcumin for 24 h, 15 *μ*M curcumin for 24 h of incubation plus hemin and hemin alone. A significant increase in Nrf2 activity was seen when curcumin was present (Figure 7(c), *P* < 0.05), which is consistent with the immunocytochemical data (Figures 7(a) and 7(b)). COS-7 cells transfected with Nrf2 were used as a positive control (Figure 7(c), last column) of the assay. The activity of this positive control showed the reliability of this assay.

### 3.6. Curcumin Pretreatment Induces the Activity of GR, GST, and SOD in CGNs

**Figure 8:**
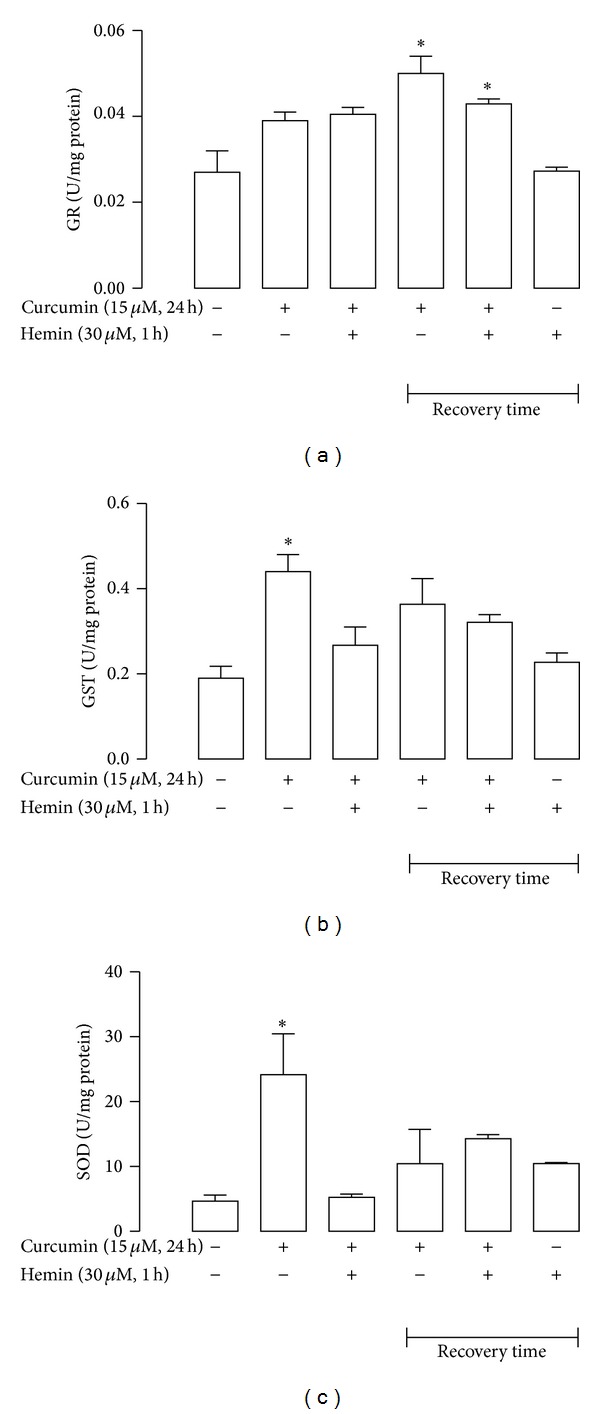
Curcumin increased activity of antioxidant enzymes in CGNs. (a) Glutathione reductase (GR), (b) glutathione-S-transferase (GST) and (c) superoxide dismutase (SOD). CGNs were treated with 15 *μ*M curcumin for 24 h. The culture medium was removed and then 30 *μ*M hemin was added for 1 h. Hemin was subsequently replaced by fresh medium and the incubation was continued. Recovery time 24 h after exposition to hemin or curcumin was considered (see legend to Figure 7). Data are expressed as mean ± SEM, *n* = 3–5. *P < 0.05 versus control (untreated).

The activity of the antioxidant enzymes was measured in cells incubated for 24 h with 15 *μ*M curcumin and treated with 30 *μ*M hemin for 1 h. Additionally, enzyme activity was determined 24 h after (recovery time) both compounds were removed. Activity of GR, GST, and SOD was increased in CGNs treated with curcumin for 24 h (Figure 8). GR activity was significantly increased in the recovery time after incubation with curcumin or curcumin/hemin (Figure 8(a), *P* < 0.05). In addition, the activity of GST and SOD was only significantly increased after 24 h of curcumin incubation (*P* < 0.05). A nonsignificant increase of the activity of GST and SOD was observed with curcumin or cotreatment with curcumin/hemin with recovery time of 24 h after incubation (Figures 8(b) and 8(c)).

## 4. Discussion

The purpose of this work was to study the effect of curcumin against the hemin-induced damage in CGNs. Protection by curcumin of different cells in culture and rat models against a variety of damages has been previously described [28, 37–39]; however, no studies have tested the potential protective effect of curcumin against hemin toxicity in neurons.

It has been described in CGNs that hemin is toxic by itself. First, hemin can be accumulated in neurons (in part by heme carrier protein 1) and secondly, Fe^3+^ was not the main effector of the damage because the use of iron chelators was unable to prevent hemin toxicity. Also, HO-1 expression was not augmented in CGNs [24]. Our data also show hemin toxicity, in fact an increase of ROS production and cellular damage was shown after 1 h of incubation with hemin. HO-1 expression was not significantly increased with hemin at the time and concentrations used in this work (data not shown). On the other hand, it has been known that curcumin can provide neuroprotection via ROS scavenging, iron chelation, modulation of cell-signaling pathways, and inhibition of inflammation [38, 40]. Moreover, curcumin is able to cross the blood-brain barrier and is neuroprotective in neurological disorders. Several studies in different experimental models of Parkinson's and Alzheimer's diseases strongly support the clinical application of curcumin in these pathologies [41, 42].

Based on the above data, it was found that curcumin was not toxic to CGNs in concentrations below 50 *μ*M when incubated for 24 h. Also, only pretreatment of curcumin prevented hemin-induced cell death, and the cotreatment with curcumin/hemin failed to protect neurons. It was found that curcumin was effective to prevent oxidative damage in cultured neurons only when it was added as a pretreatment. Interestingly, this has been observed in rats with potassium dichromate-induced nephrotoxicity [28] but not in rats with 5/6 nephrectomy in which the curcumin posttreatment was effective to reverse renal damage [5]. This may suggest that posttreatment is effective only in some experimental in vivo studies.

Additionally, curcumin alone was able to slightly raise ROS production after 24 h of incubation, but this polyphenol was capable of blocking hemin-induced ROS formation. In this context, curcumin has been considered as a hormetin, because it is an inductor of mild stress-induced of pathways of protection, maintenance, and repair [26, 27, 43].

Curcumin may exert protective effects acting either as direct antioxidant or indirect antioxidant. ROS scavenging capacity of curcumin (direct antioxidant effect) is mainly attributed to its structure as a bis-*α*,*β*-unsaturated *β*-diketone of two ferulic acid units, connected through a methylene group, and in addition, curcumin can modify the thiol groups of Keap1 releasing Nrf2 that migrates to the nucleus and induces the expression of antioxidant enzymes (indirect antioxidant effect) [1].

In the present study, curcumin induced HO-1 in CGNs in a concentration and time-dependent manner. In fact, it has been described that curcumin can raise HO-1 levels in different organs and models as in renal epithelial cells, rat hippocampal neurons, astrocytes, and normal human skin fibroblast [18, 27, 44, 45]. It was found that the protective effect of curcumin was blocked with SnMP suggesting a role of HO in the protective function, in CGNs. There are evidences that the products of HO reaction biliverdin (quickly converted into bilirubin by biliverdin reductase) and CO could give protection in cerebral vessels and CGNs [29, 31, 46]. However, SnMP was unable to exacerbate hemin-induced cell death. These findings suggest that curcumin induces the expression of HO-1 that is a part of the complete antioxidant response of the cells, which is involved in the cytoprotection.

The brain maintains a redox balance in oxidative conditions by increasing GSH levels that attenuates cell damage or death. GSH is synthesized by neurons and glial cells and is the most abundant soluble antioxidant molecule in the brain [47, 48]. In addition, GSH protects significantly neurons in vitro from oxidative condition induced by 6-hydroxydopamine, N-methyl-4-phenylpyridinium ion and dopamine [49, 50].

Curcumin increased significantly GSH levels in CGNs. Furthermore, hemin increased GSSG levels that were prevented by curcumin pre-treatment. Hemin can interact with GHS and thus prevents association of hemin with red cell membrane [51]. In addition, BSO was able to avoid protection on cell viability in cotreatment with curcumin and hemin. These results agree with those found in astrocytes treated with hemin suggesting a critical role for astroglial GSH in the cellular defense against oxidative stress in the brain. However, the mechanism whereby GSH limits hemin toxicity remains incompletely understood [22].

Taken these results together, we tested whether Nrf2 pathway was involved in this process, because the induction by curcumin of HO-1 and GSH synthesis and many detoxifying and cytoprotective enzymes is mediated by Nrf2 [1, 45]. Under no stress condition, Nrf2 controls basal expression of its target genes and is continually targeted by Keap1 for degradation catalyzed by the 26S proteasome via the ubiquitin-dependent way [52].

It has been shown that curcumin induces Nrf2 in a variety of models [4, 5, 27, 45, 53, 54]. Curcumin is an effective activator of the Keap1-Nrf2 pathway because it is a double Michael acceptor that contains two acceptor groups and is able to form conjugates with two thiol groups [14]. It was found that curcumin activates and translocates to nuclear localization of Nrf2 in CGNs. Nrf2 was readily detectable after 4 h and persisted over a period of at least 24 h of exposure, with apparent maximal amounts of Nrf2 observed after 16 h of exposure. In regard to time, curcumin showed maximal activation of Nrf2 from 8 to 48 h in diverse models [27, 37, 54]. Also with the flavonoid quercetin, nuclear location of Nrf2 was seen with 24 h of incubation in CGNs [55]. This suggests that over the time metabolism of phytochemical compounds is significant in generating mild stress which activates Nrf2.

In addition, we also evaluated the effect of curcumin in CGNs on enzyme activity of GR, GST, and SOD that are cytoprotective enzymes regulated by the Nrf2 pathway. Our results demonstrate that curcumin was able to increase the activity of these enzymes and pretreatment before the addition of hemin was capable of augmenting it. In neurons, the amount of GR is enough to allow the quick reduction of the accumulated GSSG. Under oxidative stress, GR maintains the equilibrium of the [GSH]/[GSSG] redox state in the cell [56]. Several xenobiotics react with GST to form GSH conjugation, leading to detoxication of these compounds and their excretion from the cell [47]. SOD catalyzes the dismutation of superoxide radicals into H_2_O_2_ which is then converted into water by catalase, glutathione peroxidase, or peroxiredoxin [21]. This is related with hemorrhagic stroke, because injury is reduced in mice overexpressing SOD, including diminished expression of inducible nitric oxide synthase within the cerebral cortex and attenuation of peroxidative damage [57, 58].

## 5. Conclusions

Our data suggest that the pretreatment with curcumin induces Nrf2 and an antioxidant response that may be involved in the protective effect of this antioxidant against hemin-induced neuronal death.
